# *Cytauxzoon europaeus* infections in domestic cats in Switzerland and in European wildcats in France: a tale that started more than two decades ago

**DOI:** 10.1186/s13071-021-05111-8

**Published:** 2022-01-08

**Authors:** Barbara Willi, Marina L. Meli, Chiara Cafarelli, Urs O. Gilli, Anja Kipar, Alina Hubbuch, Barbara Riond, Judith Howard, Daniel Schaarschmidt, Walter Regli, Regina Hofmann-Lehmann

**Affiliations:** 1 Clinic for Small Animal Internal Medicine, Department for Small Animals, Vetsuisse Faculty, University of Zurich, Zurich, Switzerland; 2 Clinical Laboratory, Department of Clinical Diagnostics and Services, Vetsuisse Faculty, University of Zurich, Zurich, Switzerland; 3 Center for Clinical Studies, Vetsuisse Faculty, University of Zurich, Zurich, Switzerland; 4 IDEXX Diavet Laboratories, Bäch, Switzerland; 5 Institute of Veterinary Pathology, Vetsuisse Faculty, University of Zurich, Zurich, Switzerland; 6 Clinical Diagnostic Laboratory, Department of Clinical Veterinary Medicine, Vetsuisse Faculty, University of Bern, Bern, Switzerland; 7 Labor Am Zugersee, Hünenberg, Switzerland; 8 Labor Zentral, Geuensee, Switzerland

**Keywords:** *Cytauxzoon* spp., *Cytauxzoon* sp., *Cytauxzoon felis*, Domestic cats, Stray cats, Wild felids, European wildcat, Phylogenetic analysis, *18S* rRNA, Prevalence

## Abstract

**Background:**

*Cytauxzoon* spp. infection is believed to be a newly emerging tick-borne disease in felids in Europe, with three species of the haemoparasite having recently been differentiated in wild felids. In Switzerland, rare infections have been documented in domestic cats in the west and northwest of the country, the first of which was in 2014. The aims of the present study were: (i) to characterize a *Cytauxzoon* spp. hotspot in domestic cats in central Switzerland; (ii) to elucidate the geographic distribution of *Cytauxzoon* spp. in domestic cats in Switzerland; (iii) to assess suspected high-risk populations, such as stray and anaemic cats; and (iv) to investigate the newly emerging nature of the infection. *Cytauxzoon* spp. were further differentiated using mitochondrial gene sequencing.

**Methods:**

The overall study included samples from 13 cats from two households in central Switzerland (study A), 881 cats from all regions of Switzerland (study B), 91 stray cats from a hotspot region in the northwest of Switzerland and 501 anaemic cats from across Switzerland (study C), and 65 Swiss domestic cats sampled in 2003 and 34 European wildcats from eastern France sampled in the period 1995–1996 (study D). The samples were analysed for *Cytauxzoon* spp. using real-time TaqMan quantitative PCR, and positive samples were subjected to *18S* rRNA, cytochrome *b* (*CytB*) and cytochrome *c* oxidase subunit I (*COI*) gene sequencing.

**Results:**

In study A, six of 13 cats from two neighbouring households in central Switzerland tested postive for *Cytauxzoon* spp.; two of the six infected cats died from bacterial infections. In studies B and C, only one of the 881 cats (0.1%; 95% confidence interval [CI]: 0–0.3%) in the countrywide survey and one of the 501 anaemic cats (0.2%; 95% CI: 0–0.6%) tested postive for *Cytauxzoon* spp. while eight of the 91 stray cats in the northwest of Switzerland tested positive (8.8%; 95% CI: 3.0–14.6%). In study D, *Cytauxzoon* spp. was detected in one of the 65 domestic cat samples from 2003 (1.5%; 95% CI: 0–4.5%) and in ten of the 34 European wildcat samples from 1995 to 1996 (29%; 95% CI: 14.2–44.7%). The isolates showed ≥ 98.6% sequence identities among the *18S* rRNA, *CytB* and *COI* genes, respectively, and fell in the subclade *Cytauxzoon europaeus* based on *CytB* and *COI* gene phylogenetic analyses.

**Conclusions:**

The study challenges the newly emerging nature of *Cytauxzoon* spp. in central Europe and confirms that isolates from domestic cats in Switzerland and European wild felids belong to the same species.

**Graphical Abstract:**

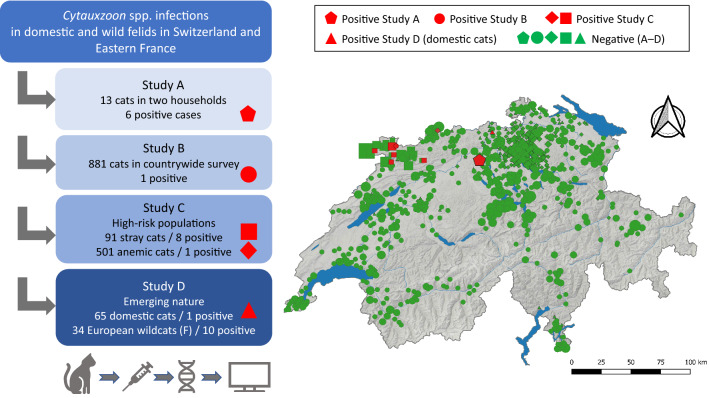

**Supplementary Information:**

The online version contains supplementary material available at 10.1186/s13071-021-05111-8.

## Background

Cytauxzoonosis is a tick-borne disease of domestic cats and wild felids that is caused by the apicomplexan haemoparasites *Cytauxzoon* spp. *Cytauxzoon felis* is the best-characterized species within this group of pathogens [1, 2]. Infection generally induces a rapidly progressive disease in domestic cats that is associated with high mortality [3, 4], but survival after clinical infection and subclinical persistent parasitaemia has been documented [5–7]. Since the first report in the mid-1970s [8], *C. felis* infection has been considered an emerging disease with an expanding case distribution in the USA [9]. Both *Dermacentor variabilis* and *Amblyomma americanum* ticks are assumed to transmit *C. felis* [10, 11], but more recent studies point to *A. americanum* as the primary definitive host and tick vector of *C. felis* [10, 12–14]. The main natural reservoir of *C. felis* in the USA is considered to be the bobcat (*Lynx rufus*). Infections in this species are usually subclinical [11, 15–17], but fatal disease has been described in bobcats, tigers and lions [15, 18–21].

In recent years, *Cytauxzoon* spp. infections with a species molecularly distinct from *C. felis* have been reported in domestic cats in a number of European countries, including Italy, France, Spain, Portugal and, most recently, Switzerland and Germany [22–30]. There have also been reports of infections with *Cytauxzoon* spp. in wild felids in Europe; these include the European wildcat in Italy, Romania, Germany, Luxembourg, Bosnia and Herzegovina [31–34], the Iberian lynx in Spain [35] and the Eurasian lynx in Romania, Czech Republic and Switzerland [32, 34] (MLM, personal communication). Similar to how bobcats serve as the primary reservoir of *C. felis* in the USA [3], the Eurasian and the Iberian lynx may play a role as reservoirs of *Cytauxzoon* spp. in Europe. The role of the European wildcat in the transmission cycle of *Cytauxzoon* spp. is unclear, and the tick vector involved in the transmission of *Cytauxzoon* spp. in Europe has not yet been identified.

Based on sequencing of the *18S* rRNA gene, *Cytauxzoon* spp. isolates from Europe are phylogenetically related but distinct from *C. felis* and most closely related to *Cytauxzoon manul* from the Pallas’ cat [36, 37]. More recently, Panait et al. [34] suggested that three distinct species of *Cytauxzoon* exist in European wild felids, based on results of phylogenetic analyses of the two mitochondrial genes cytochrome *b* (*CytB*) and cytochrome *c* oxidase subunit I (*COI*) from *Cytauxzoon* spp. isolates obtained from European wildcats and Eurasian lynx from different European countries. One species predominated (*Cytauxzoon europaeus*), while the two other species were rare and found only in seven (*Cytauxzoon otrantorum*) and one (*Cytauxzoon banethi*) European wildcats in Romania, respectively [34]. Whether these species also occur in domestic cats is currently unknown.

*Cytauxzoon* spp. infections in domestic cats in Europe are generally considered to be less severe than *C. felis* infections. Indeed, in one study, 22.9% of the investigated cats in northeast Italy tested PCR positive for *Cytauxzoon* spp. even though most of these cats appeared to be clinically healthy [23]. Nevertheless, symptomatic and fatal *Cytauxzoon* spp. infections have been described in domestic cats in Europe [22, 23, 26, 27, 29], with these cats presenting with lethargy, anorexia, weight loss, pyrexia, pale mucous membranes, diarrhoea, vomiting and pleural or peritoneal effusion; however, concomitant diseases were not always excluded [23, 26].

Recently, we reported *Cytauxzoon* spp. infection for the first time in Switzerland in five domestic cats [29]. All infected cats were from the northwest and west of Switzerland, regions close to the French border. Three infected kittens from the same litter presented with moderate to severe anaemia. The kittens recovered following treatment with atovaquone and azithromycin but developed long-term asymptomatic parasitaemia. One adult cat with non-regenerative anaemia experienced accidental transmission of *Cytauxzoon* spp. through a blood transfusion from an asymptomatically infected cat.

The recent discovery and expanding geographical distribution of *Cytauxzoon* spp. in domestic cats and wild felids in Europe suggest that *Cytauxzoon* spp. infection is an emerging tick-borne disease, but this hypothesis has not been investigated. Following our initial report of *Cytauxzoon* spp. infection in cats in west and northwest Switzerland in 2014/2015, we discovered an additional six cases in domestic cats in Switzerland that were diagnosed in 2019 (study A); these cats originated from two households in central Switzerland. This finding prompted us to further elucidate the epidemiology and emerging nature of *Cytauxzoon* spp. infection in Switzerland. For this purpose, we conducted a countrywide survey in privately owned cats from each canton (region) in Switzerland (study B) and investigated the frequency of infection in suspected high-risk populations, such as anaemic cats in Switzerland and stray cats in northwestern Switzerland (study C). To address the question of whether *Cytauxzoon* spp. infection has newly emerged in domestic and wild felids in central Europe, we tested DNA from blood of domestic cats and European wildcats collected in 2003 and 1995–1996, respectively, for the presence of the organism (study D). All *Cytauxzoon* spp. isolates were subjected to *18S* rRNA and mitochondrial gene amplification in order to perform in-depth phylogenetic analyses and resolve the genetic relationship of the *Cytauxzoon* spp. isolates from domestic and wild felids.

## Methods

### Study design

The present study comprised four substudies (studies A–D). In study A, we reported the occurrence of *Cytauxzoon* spp. infection in six cats from two households in central Switzerland. Study B addressed the countrywide occurrence of *Cytauxzoon* spp. infection in domestic cats in Switzerland. In study C, we investigated *Cytauxzoon* spp. infection in suspected high-risk populations, i.e. stray cats in a hotspot region in Switzerland and anaemic cats in Switzerland. Study D addressed the potential emerging nature of *Cytauxzoon* spp. infections in domestic cats and wild felids in central Europe. Phylogenetic analysis of the *Cytauxzoon* spp. isolates was performed based upon sequencing of the *18S* rRNA gene and two mitochondrial genes (*CytB* and *COI*). Samples for studies B and D and the samples from stray cats for study C were available from previous studies [38–41].

### Sample characteristics

In study A, 13 cats were tested for *Cytauxzoon* spp. infection (Table 1). The diagnostic services of the Clinical Laboratory, Vetsuisse Faculty Zurich first identified three cats infected with *Cytauxzoon* spp. in a household in central Switzerland in February–March 2019 (Canton Aargau, household 1) and subsequently in three cats in May 2019 in a neighbouring household in the same village (household 2); in household 2, seven cats subsequently underwent diagnostic testing. Data on the place and region of origin, age, sex and general health status were available for all cats from households 1 and 2 (Additional file 1: Table S1), and all cats were tested for *Cytauxzoon* spp. and feline leukaemia virus (FeLV) provirus by real-time TaqMan quantitative PCR (qPCR) and for feline immunodeficiency virus (FIV) by western blot (Table 2; Additional file 1: Table S1) [42, 43].

**Table 1:** Characteristics of the samples from domestic cats and European wildcats included in the study

Study	Country	Species	Lifestyle/origin	Year(s) of sample collection	Region of origin (canton^a^ or department)	No. of animals	Sex, *n* (%)					Purebred, *n* (%)			Reference
							Male intact	Male castrated	Female intact	Female castrated	Not known	Yes	No	Not known	
A	Switzerland	Domestic cats	Privately owned	2019	AG	13	0	12 (92.3%)	0	1 (7.7%)	0	0	13 (100%)	0	This study
B	Switzerland	Domestic cats	Presented to veterinarians	2013–2016	All 26 cantons	881	56 (6.4%)	308 (35.0%)	70 (7.9%)	207 (23.5%)	240 (27.2%)	160 (18.2%)	479 (54.4%)	242 (27.5%)	[39]
C	Switzerland	Domestic cats	Presented to veterinarians	2019–2021	AG, BE, BL, GL GR, LU, NW, OW, SG, SH, SO, SZ, TG, VD, ZG, ZH	501	0	0	0	0	501	0	0	501	This study
C	Switzerland	Domestic cats	Stray	2014	JU	91	37 (40.7%)	0	48 (52.7%)	0	6 (6.6%)	0	91 (100%)	0	[38]
D	Switzerland	Domestic cats	Presented to veterinarians	2003	AG, BE, BL, FR, JU, LU, NE, SH, SO, TG, TI, VD, ZH	65	2 (3.1%)	32 (49.2%)	1 (1.5%)	24 (36.9%)	6 (9.2%)	47 (72.3%)	12 (18.5%)	6 (9.2%)	[40]
D	France	European wildcats	Free ranging	1995–1996	Haute-Marne, Marne, Vosges, Aube, Meurthe-et-Moselle	34	19 (55.9%)	0	15 (44.1%)	0	0	NA	NA	NA	[41]

NA, Not applicable

^a^AG, Aargau; BE, Bern; BL, Basel-Land; FR, Fribourg; GL, Glarus; GR, Grison; JU, Jura; LU, Lucerne; NE, Neuchâtel; NW, Nidwalden; OW, Obwalden; SG: St. Gallen; SH, Schaffhausen; SO, Solothurn; SZ, Schwyz; TG, Thurgau; TI, Ticino; VD, Vaud; ZG, Zug; ZH, Zurich

**Table 2:** Retrovirus status and *Cytauxzoon* spp. PCR test results in domestic cats and European wildcats in studies A–D

Study	Samples, year of collection	Number of samples	FeLV status, *n* (%; 95% CI)			FIV antibody-positive^a^, *n* (%; 95% CI)	PCR-positive test results for *Cytauxzoon* spp.^b^, *n* (%; 95% CI)
			Provirus positive		p27 antigen positive		
A	Domestic cats, 2019	13	1 (7.7%; 0–22.2%)		ND	4 (30.8%; 5.7–55.9%)	6 (46.1%; 19.1–73.3%)
B	Domestic cats, 2013–2016	881	47 (5.3%; 3.9–6.8%)		18 (2.0%; 1.2–3.2%)	ND	1 (0.1%; 0–0.3%)
C	Domestic cats, 2019–2021	501	ND		ND	ND	1 (0.2%; 0–0.6%)
C	Stray domestic cats, 2014	91	7 (7.7%; 2.2–13.2%)		ND	ND	8 (8.8%; 3.0–14.6%)
D	Domestic cats, 2003	65	ND		3 (6.4%; 0–13.4%)^c^	2 (4.5%; 0–10.7%)^d^	1 (1.5%; 0–4.5%)
D	European wildcats, 1995–1996	34	ND		26 (76.5%; 62.2–90.7%)	0^e^	10 (29.4%; 14.1–44.7%)

CI, Confidence interval; ND, not determined

^a^Determined by FIV ELISA; positive results confirmed by western blot analysis

^b^Positive by real-time TaqMan quantitative PCR and/or conventional PCR test results and confirmed by sequencing

^c^FeLV p27 antigen results were not available in 18 cats

^d^FIV ELISA results were not available in 21 cats

^e^FIV antibody results were not available for 3 European wildcats

One cat from household 1 underwent a complete necropsy and subsequent histological examination of selected organs (heart, lungs, liver, pancreas, kidney, spleen, lymph nodes) at the Institute of Veterinary Pathology, Vetsuisse Faculty Zurich, Switzerland. Sections (3–4 µm) from formalin-fixed, paraffin-embedded tissue specimens were routinely stained with haematoxylin/eosin and Giemsa. Sections from the lung were also subjected to immunohistological staining for feline CD18 to highlight monocytes/macrophages [44]. Cytological specimens of the pleural effusion were examined, and real-time TaqMan qPCR for *Cytauxzoon* spp. was performed on blood, pleural fluid, lung, bone marrow, liver, kidney, spleen and pancreas at the Clinical Laboratory, Vetsuisse Faculty Zurich.

Study B comprised 881 total nucleic acid (TNA) samples extracted from ethylenediaminetetraacetic acid (EDTA)-anticoagulated blood of domestic cats presented to private veterinarians for diagnostic purposes (Table 1). The samples had been collected as part of a FeLV prevalence study between September 2013 and April 2016; information on these samples has been published in detail [38, 39]. Data on place and region (canton) of origin and sex were available for most cats (Table 1). FeLV provirus real-time TaqMan qPCR results were available from the previous study (Table 2) [39]. The quality and quantity of the TNA samples was assessed using a feline albumin real-time TaqMan qPCR assay [45], and only samples with a cycle threshold (CT) value < 30 were included.

In study C, the frequency of *Cytauxzoon* spp. infection in suspected high-risk populations was assessed by testing TNA extracted from EDTA-anticoagulated blood of 501 anaemic cats from Switzerland; blood samples were sent for diagnostic purposes to the Clinical Laboratory, Vetsuisse Faculty Zurich between 2019 and 2021 (Table 1). The samples originated from 16 cantons in Switzerland (Table 1) and included 33 cats from the canton of Aargau, where the two affected households from study part A were located; one cat came from the western part of Switzerland (canton of Vaud), where the remaining *Cytauxzoon* spp. cases in Switzerland have been documented [29]. In addition, TNA samples were included from EDTA-anticoagulated blood of 91 stray cats collected during a trap-neuter-release programme in August and October 2014 in a known hotspot region in northwestern Switzerland in the canton of Jura (Table 1); details of these samples have been published [38]. The sampled cats originated from 19 different villages in the canton of Jura in northwestern Switzerland. The samples had already been analysed for *Felis catus* gammaherpesvirus (FcaGHV) in a previous study [38], and data on sex and FeLV status were available for most animals (Tables 1, 2). All stray cat TNA samples were evaluated using a feline albumin real-time TaqMan qPCR assay [45], and only samples with a CT < 30 were included.

**Fig. 1:**
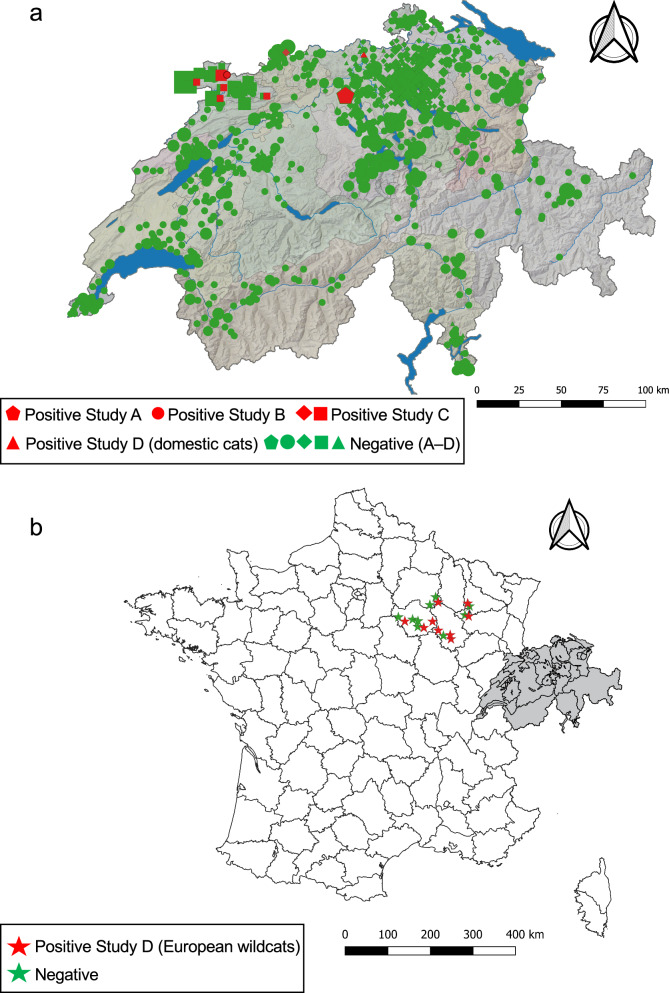
Map of Switzerland (**a**) and France (**b**) showing the geographical distribution of the analysed samples. The geographic origin of the cats from study parts A (pentagons), B (circles), C (rhombs: anaemic cats; squares: stray cats) and D (triangles: domestic cats from 2003; stars: European wildcats from 1995–1995) in Switzerland (**a**) and France (**b**) are indicated. The colour of the symbols in **a** and **b** indicate PCR-positive (red) and -negative (green) samples. The size of the symbols in **a** indicates the number of *Cytauxzoon* spp. PCR-positive or -negative samples per location. For 11 cats from study B and 30 stray cats from study C the place of origin within the canton was unknown; these samples were allocated to the capital city of the corresponding canton

In study D, the emerging nature of *Cytauxzoon* spp. infection was investigated by testing DNA samples from EDTA-anticoagulated blood of 65 privately-owned cats from Switzerland sampled in 2003 (Table 1). The samples were part of a larger set of feline blood samples previously tested for feline haemoplasmas [40]. The cats originated from the south (*n* = 8), the northwest and west (*n* = 9), and central, eastern and northern Switzerland (*n* = 48, Fig. 1a). Furthermore, DNA was included from the blood of 34 European wildcats collected between March 1995 and October 1996 for an unrelated study (Table 1) [41]; the samples originated from six different departments in eastern France (Fig. 1b). Data on place and region of origin, age/age group, sex and retrovirus status were available for most of the included cats and wild felids (Tables 1, 2). The quality and quantity of TNA were evaluated in 47/65 domestic cat samples using the feline albumin real-time TaqMan qPCR assay [45].

### Haematology and blood biochemistry

The results from haematology and blood biochemistry analyses were available for five cats infected with *Cytauxzoon* spp. from households 1 and 2 (Additional file 2: Table S2; Additional file 3: Table S3); one cat was tested twice, at initial diagnosis (March 2019) and at the time of euthanasia (May 2019; Additional file 2: Table S2; Additional file 3: Table S3). Haematology and blood biochemistry analyses were performed at the Clinical Laboratory, Vetsuisse Faculty, University of Zurich on a Sysmex XT-2000iV (Sysmex Corporation, Kobe, Japan) [46] and a Cobas C 501 instrument (Roche Diagnostics AG, Rotkreuz, Switzerland), respectively, or at IDEXX Laboratories (IDEXX Diavet AG, Bäch, Switzerland) using a Sysmex XT-2000iV (Sysmex Corporation) and an AU680 ISE (Beckman Coulter, Inc., Brea CA, USA), respectively. Laboratory (internal validation IDEXX Diavet/IDEXX Ludwigsburg, Ludwigsburg, Germany), and published reference intervals were used [46].

### TNA extraction

Total nucleic acid extraction in study A was performed from 100 μl of EDTA-anticoagulated blood using the MagNa Pure LC (Roche Diagnostics AG) and the MagNa Pure LC TNA Isolation Kit (Roche Diagnostics AG) following the manufacturer’s instructions. DNA from tissues collected upon necropsy was purified using DNeasy Tissue Kits (Qiagen, Hilden, Germay). A negative control consisting of 100 µl of phosphate buffered saline was used with each batch of extraction to monitor for cross-contamination. The TNA and DNA samples were stored at − 80 °C until PCR analysis was performed.

### Diagnostic assays

The FeLV provirus was detected using a real-time TaqMan qPCR assay as described [42]. FeLV p27 antigenaemia was determined using a published sandwich enzyme-linked immunosorbent assay (ELISA) [47]; a sample signal of ≥ 20% of the positive control signal was considered to be positive. FIV infection was detected using a western blot [48]. Published real-time TaqMan qPCR assays were used for the detection of feline albumin and *18S* ribosomal RNA (rRNA) [45].

All blood DNA and TNA samples of this study were subjected to a *Cytauxzoon* spp. real-time TaqMan qPCR assay, with the exception of the samples from European wildcats; the latter were tested directly by conventional PCR (see following section). Tissue samples were tested undiluted and at a 1:10 dilution to detect inhibition; if inhibition was present, the CT value of the diluted sample is given. The primers and probe of the real-time TaqMan qPCR assay were designed to specifically detect a 69-bp fragment of the *18S* rRNA sequences of *Cytauxzoon* spp. found in the NCBI GenBank [29]. All samples with CT values < 35 were subsequently subjected to conventional PCR to amplify 219 bp of the *18S* rRNA gene of *Cytauxzoon* spp. [29, 49, 50]. The PCR products were separated in a 2% agarose gel, and bands of the appropriate size (219 bp) were sequenced in a commercial laboratory (Microsynth AG, Balgach, Switzerland) using the amplification primers. All real-time TaqMan qPCR assays were performed on an ABI 7500 Fast Real-Time PCR system (Applied Biosystems, Thermo Fisher Scientific, Waltham, MA, USA). Positive and negative controls were run with each PCR assay and consisted of DNA from a *Cytauxzoon* spp. PCR-positive Iberian lynx (confirmed by sequencing) and of nuclease-free water, respectively.

### Amplification and sequencing of the *18S *rRNA, *CytB* and *COI* genes

All samples that were PCR positive in conventional PCR underwent amplification and sequencing of the *18S* rRNA, *CytB* and *COI* genes of *Cytauxzoon* spp. The almost complete *18S* rRNA gene was amplified using the forward primer Cytlblynx.23f (5ʹ-GCC ATG CAT GTC TAA GTA TAA GC-3ʹ) and the reverse primer Cytlblynx.1659r (5ʹ-CGC GCC TAA CGA ATT AGA AG-3ʹ) as previously described [29]. The mitochondrial *CytB* and *COI* genes were amplified from the same samples using nested PCR assays as previously published [34], with some modifications. Briefly, the reaction mixture contained 5 µl of 5×  HF PCR buffer (Finnzymes Oy, Espoo, Finland/BioConcept AG, Allschwil, Switzerland), 500 nM of each primer, 0.2 mM of each deoxynucleotide triphosphate (Sigma-Aldrich, St. Louis, MO, USA), 1 U Phusion High-Fidelity DNA Polymerase (Finnzymes Oy) and 5 µl template TNA/DNA, with water added to achieve a a final reaction volume of 25 µl. The conventional PCR assays were performed on a Biometra T-Personal 48 Thermocycler (Biometra, Gottingen, Germany), using the following cycling programme: 98 °C for 30 s; 35 cycles of 98 °C for 10 s, 53 °C (Cytaux_cytb_F1/Cytaux_cytb_R3 [*CytB*]), 55 °C (Cytaux_cytb_Finn/Cytaux_cytb_Rinn [*CytB*]), 64 °C (Th-For2/Piro_mt_R1 [*COI*]), 60 °C (Th-For2/Cytaux_260R [*COI*]) for 30 s, 72 °C for 1 min; and a final elongation at 72 °C for 10 min. PCR products were separated in a 1.5% agarose gel, and bands of the appropriate size were cut and extracted using the MinElute® Gel Extraction Kit (Qiagen) and the PCR products subsequently sequenced.

### Genetic and phylogenetic analyses

Sequences were edited and assembled using Geneious® prime software (Biomatters Limited, Auckland, New Zealand) [51]. Phylogenetic analysis was conducted using MEGA-X [52]. Sequences were aligned to additional reference sequences retrieved from GenBank using the Clustal W algorithm [53]. The phylogenetic tree was inferred using the maximum likelihood method using a distance matrix corrected for nucleotide substitutions based on the Kimura 2-parameter model [54]. The initial tree(s) for the heuristic search were automatically obtained by applying neighbour-joining and BioNJ algorithms to a matrix of pairwise distances estimated using the maximum composite likelihood approach, and then selecting the topology with superior log likelihood value. Codon positions included were 1st + 2nd + 3rd + noncoding. All positions containing gaps and missing data were eliminated. The dataset was resampled 1000 times to generate bootstrap values [55].

### Statistical analysis

For the observed sample prevalences, 95% confidence intervals (CIs) were calculated using XLSTAT for Microsoft Excel (Addinsoft, NY, USA). The association between *Cytauxzoon* spp. infection and exposure variables was tested by univariate statistical analysis performed by a chi-squared (*χ*^2^) test or a Fisher’s exact test for small numbers (*n* < 5) using XLSTAT (Addinsoft). *P* values < 0.05 were considered to be statistically significant.

## Results

### Study A:* Cytauxzoon* spp. infection in six cats from two neighbouring households in Switzerland

After the first report of *Cytauxzoon* spp. infection in five domestic cats from northwestern and western Switzerland in 2014/1015 [29], the organism was detected again in a cat that was presented to a private veterinary practitioner in February 2019 and subsequently in another five cats based on PCR analysis of TNA extracted from blood samples sent to the Clinical Laboratory, University of Zurich for testing. These cats came from two households in central Switzerland (canton of Aargau), an area where *Cytauxzoon* spp. had not been previously reported (Table 1; Fig. 1a). The two neighbouring households were located only 182 m apart. A total of 13 cats were investigated, of which six (46.1%; 95% CI: 19.1–73.3%) were infected with *Cytauxzoon* spp. (Table 2). All cats were born in the region and had never travelled abroad.

*Cytauxzoon* spp. infection was first detected in a cat from household 1 that was taken to a private veterinary practitioner due to severe and progressive cellulitis on the right front limb (Additional file 1: Table S1). EDTA-anticoagulated blood and serum were taken 1 day after the first presentation and sent to an external reference laboratory (IDEXX Diavet AG) for an extensive check-up. Blood work revealed nonregenerative anaemia, severe leukocytosis, moderate thrombocytopenia, mild hypoalbuminaemia, a mild increase in alanine aminotransferase and a moderate increase in aspartate aminotransaminase (Additional file 2: Table S2, Additional file 3: Table S3). Ring-shaped single or paired inclusion bodies were detected in the blood smear, raising suspicion for the presence of *Cytauxzoon* spp. organisms. The sample was then transferred to the Clinical Laboratory, University of Zurich, where infection with *Cytauxzoon* spp. was diagnosed from PCR analyses of TNA extracted from this blood sample and subsequently confirmed by sequencing (Additional file 1: Table S1). Retrovirus testing revealed co-infection with FIV (Additional file 1: Table S1). The cat was euthanised 3 days after first presentation due to poor prognosis; necropsy was not performed.

The two other cats from household 1 were subsequently also tested for *Cytauxzoon* spp. and retrovirus infection in March 2019. Both cats had positive PCR test results for *Cytauxzoon* spp., one of which was co-infected with FIV (Additional file 1: Table S1). At the time of diagnosis, both cats were clinically healthy, and had no blood abnormalities except for mild hyperproteinaemia in the FIV-infected cat (Additional file 2: Table S2; Additional file 3: Table S3). One of the two cats developed severe apathy and was euthanised with suspected pyothorax in May 2019 (Additional file 1: Table S1). At the time of euthanasia, the cat showed severe hyperbilirubinaemia in the absence of anaemia (Additional file 2: Table S2; Additional file 3: Table S3). The animal was subjected to a full post-mortem examination, which revealed a severe pyothorax with pulmonary atelectasis and acute fibrinosuppurative pleuritis. A cytological examination of the fluid revealed the presence of filamentous bacteria compatible with *Actinomycetaceae*, consistent with a diagnosis of actinomycosis/nocardiosis. In addition to pleuritis and atelectasis, histological examination of the lung revealed filamentous Gram-positive bacteria (consistent with *Actinomycetaceae*) in some capillaries, indicating bacteraemia. Immunohistology for the adhesion molecule CD18 stain identified rare individual large, vacuolated, strongly positive monocytes within capillaries and a few small aggregates of equally positive macrophages within alveolar lumina (Additional file 4: Figure S1a). The spleen and lymph node showed severe lymphocyte depletion, with the splenic red pulp being poorly cellular and the lymph node exhibiting very rare large vacuolated macrophages in the marginal sinuses. The heart, liver and kidney were unremarkable except for very rare, individual large vacuolated cells within the glomerular tufts and one structure suggestive of a large vacuolated monocyte in a myocardial capillary (Additional file 4: Figure S1b). These, as well as the strongly CD18-positive monocytes/macrophages, were considered indicative of the schizont-bearing monocytes/macrophages characteristic for *C. felis* infection [44, 56]; however, particularly in the lungs these could also represent cells activated due to the actinomycosis/nocardiosis. The pleural effusion and all tissue samples examined (spleen, lung, bone marrow, kidney, pancreas, liver) yielded PCR-positive test results for *Cytauxzoon* spp. but with lower loads (CT values: 24.7–30.6) compared to blood loads at the time of euthanasia (CT value: 14.7).

In April and May 2019, another 10 cats from the neighbouring household 2 were tested for *Cytauxzoon* spp. and retrovirus infection as part of health checks and because the owner indicated that the cats had aggressive interactions with the cats from household 1 (Additional file 1: Table S1). Of these 10 cats, three tested PCR positive for *Cytauxzoon* spp. and two were FIV positive; the results for FIV in the other cats were questionable according to the western blot test (detection of only 1 band). Besides some unrelated health problems, the cats were clinically healthy. Blood test results were available for two of the three *Cytauxzoon* spp.-infected animals and revealed only mild and unspecific changes (Additional file 2: Table S2; Additional file 3: Table S3). One cat had mild hyperproteinaemia, but the cat was co-infected with FIV (Additional file 1: Table S1).

### Study B: Countrywide survey on *Cytauxzoon* spp. infection in domestic cats in Switzerland

Only one of 881 domestic cat samples (0.1%; 95% CI: 0–0.3%) in the countrywide survey tested PCR positive for *Cytauxzoon* spp. (Table 2). The positive result was confirmed by sequencing (Additional file 5: Table S4). The cat came from the canton of Jura in the northwest of Switzerland (Fig. 1a). The cat tested negative for FeLV provirus and PCR negative for the three feline haemoplasmas, but had a positive PCR test result for FcaGHV-1 and had a questionable result for FIV by western blot (detection of only 1 band) [38]. No health status data were available for this cat.

### Study C: *Cytauxzoon* spp. infections in stray cats in northwestern Switzerland and in anaemic cats

Of the 501 anaemic cats, one (0.2%; 95% CI: 0–0.6%) tested *Cytauxzoon* spp. PCR positive in blood (Table 2). This cat came from northern Switzerland (canton of Basel-Land), where *Cytauxzoon* spp. had not been previously reported (Fig. 1a) and had been taken to a private veterinary practitioner because of severe anaemia (haematocrit: 12%). The cat suffered from chronic renal failure and was co-infected with FIV (Additional file 5: Table S4); no further data or follow-up of this cat were available.

Of the 91 stray cats, eight (8.8%; 95% CI 3.0–14.6%) tested positive for *Cytauxzoon* spp. (Table 2); positive results were confirmed by sequencing (Additional file 5: Table S4). The PCR-positive cats were from four different villages (Fig. 1a, Additional file 5: Table S4); the place of origin was unknown for one cat. All eight cats infected with *Cytauxzoon* spp. tested negative for FeLV provirus (Additional file 5: Table S4); no data were available on the health and FIV status of these cats.

### Study D: *Cytauxzoon* spp. infections in cats and European wildcats sampled in 2003 and 1995–1996, respectively

To investigate the emerging nature of *Cytauxzoon* spp. infection in felids, we examined samples collected more than two decades ago from domestic cats in Switzerland (*n* = 65) and European wildcats (*n* = 34) from eastern France. Of the 65 blood DNA samples collected from domestic cats in Switzerland in 2003, one (1.5%; 95% CI: 0–4.5%) tested PCR positive for *Cytauxzoon* spp. (Table 2); the result was confirmed by sequencing (Additional file 5: Table S4). This *Cytauxzoon* spp.-positive cat was from the canton of Aargau in central Switzerland (Fig. 1a) and tested negative for FeLV by FeLV p27 ELISA and negative for FIV by western blot (Additional file 5: Table S4). The cat had been taken to a veterinary clinic in 2003 because of severe anaemia and icterus. Hepatic lipidosis was diagnosed, and the cat was euthanised due to continuous deterioration. A post-mortem examination was not performed.

Of the 34 European wildcats from eastern France, 10 (29%; 95% CI: 14.2–44.7%) tested PCR positive for *Cytauxzoon* spp. (Table 2); the results were confirmed by sequencing (Additional file 6: Table S5). Eight of these 10 infected cats tested positive for FeLV p27; none were positive for FIV by western blot (Additional file 6: Table S5). The 10 European wildcats infected with *Cytauxzoon* spp. were from four different departments in eastern France (Fig. 1b; Additional file 6: Table S5); the places of origin were up to 156 km apart. Because the samples had been collected from animals hit by cars, no data on the health status of these animals were available.

### No association of *Cytauxzoon* spp. infection with sex and retrovirus status of the cats

In study C, the prevalence of *Cytauxzoon* spp. infection was higher in stray cats in the hotspot area (8.8%; 95% CI: 3.0–14.6%) than in domestic cats in the countrywide survey (0.1%; 95% CI: 0–0.3%) and in anaemic cats (0.2%; 95%: CI 0–0.6%; Table 2). Sex and retrovirus infections were not significantly associated with *Cytauxzoon* spp. infection in any group of samples.

### *Cytauxzoon* spp. sequencing results and phylogenetic analyses

**Fig. 2:**
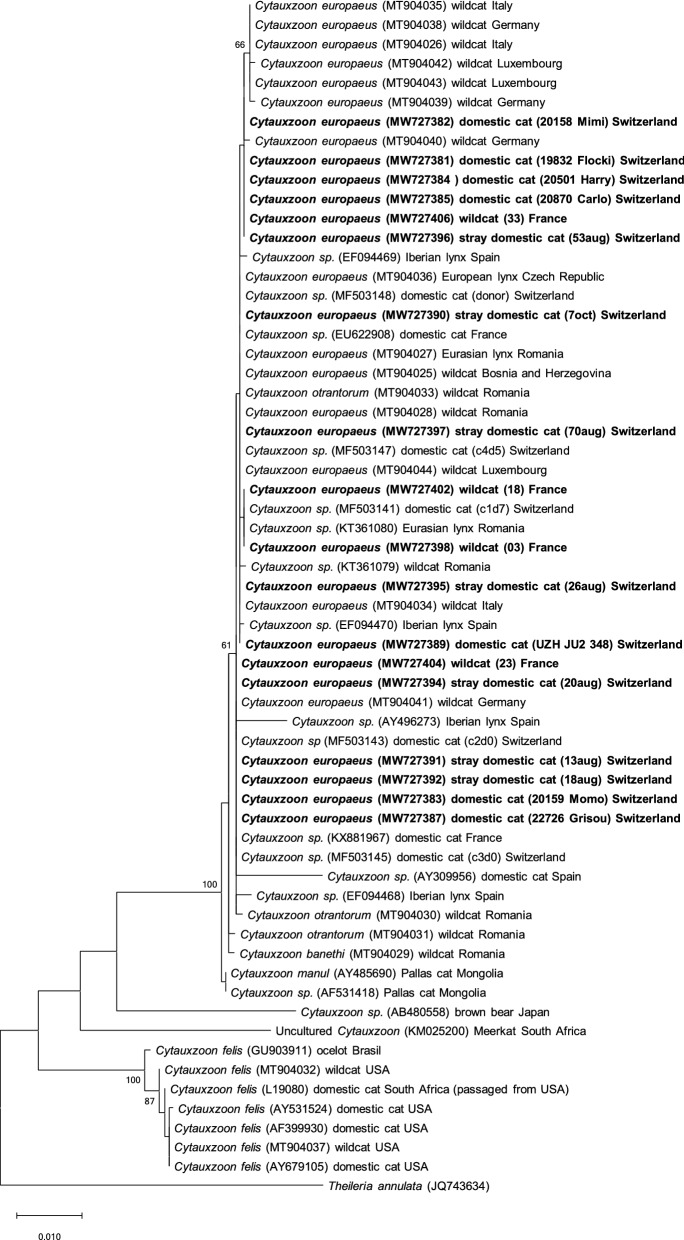
Molecular phylogenetic analysis by maximum likelihood method of the *18S* rRNA gene. The evolutionary history was inferred by using the maximum likelihood method based on the Kimura 2-parameter model [54]. The tree with the highest log likelihood (− 4029.57) is shown. The percentage of trees in which the associated taxa clustered together is shown next to the branches (values < 60% are not shown). Initial tree(s) for the heuristic search were obtained automatically by applying the neighbour-joining and BioNJ algorithms to a matrix of pairwise distances estimated using the maximum composite likelihood (MCL) approach, and then selecting the topology with superior log likelihood value. The tree is drawn to scale, with branch lengths measured in the number of substitutions per site. Host and country origin of the sequences are indicated. GenBank accession numbers are shown in brackets. Isolates from this study are written in bold font

Sequencing of the almost complete *18S* rRNA gene was successful in 18 of the 27 PCR-positive samples; from the remaining nine PCR-positive samples, a shorter fragment (219 bp) corresponding to the amplicon length of the conventional PCR was sequenced (Additional file 1: Table S1; Additional file 5: Table S4; Additional file 6: Table S5). Almost complete *18S* rRNA sequences were obtained, in particular from samples with low CT values (< 28; Additional file 1: Table S1; Additional file 5: Table S4). Sequence identities in the *18S* rRNA gene among isolates in this study ranged from 99.3 to 100%, with no clear differences between sequences from the different groups. Overall, the isolates showed < 96.5% sequence identity in the *18S* rRNA gene when compared to all *C. felis* isolates considered and > 99.2% identity with all European *Cytauxzoon* spp. and* C. manul* isolates considered (Fig. 2), with the exception of one *Cytauxzoon* spp. isolate from a Japanese brown bear (AB480558) for which the identity ranged between 89.4 and 90.6%; sequence identities were independent of host, collection date and region of origin of the samples.

**Fig. 3:**
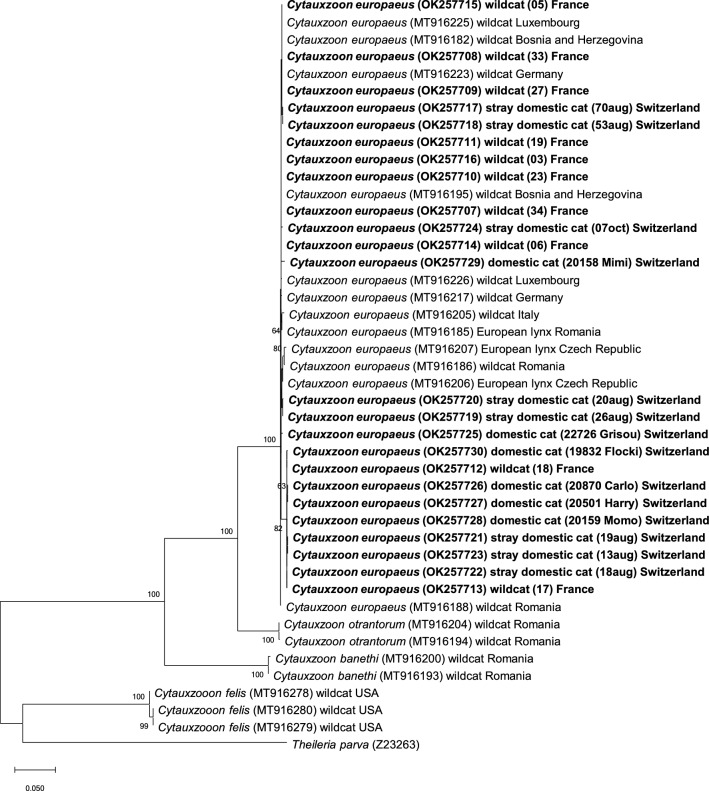
Molecular phylogenetic analysis by maximum likelihood method of the mitochondrial gene *CytB*. The evolutionary history was inferred by using the maximum likelihood method based on the Kimura 2-parameter model [54]. The tree with the highest log likelihood (− 5012.83) is shown. The percentage of trees in which the associated taxa clustered together is shown next to the branches (values < 60% are not shown). Initial tree(s) for the heuristic search were obtained automatically by applying the neighbour-joining and BioNJ algorithms to a matrix of pairwise distances estimated using the MCL approach, and then selecting the topology with superior log likelihood value. The tree is drawn to scale, with branch lengths measured in the number of substitutions per site. Host and country origin of the sequences are indicated. GenBank accession numbers are shown in brackets. Isolates from this study are written in bold font

**Fig. 4:**
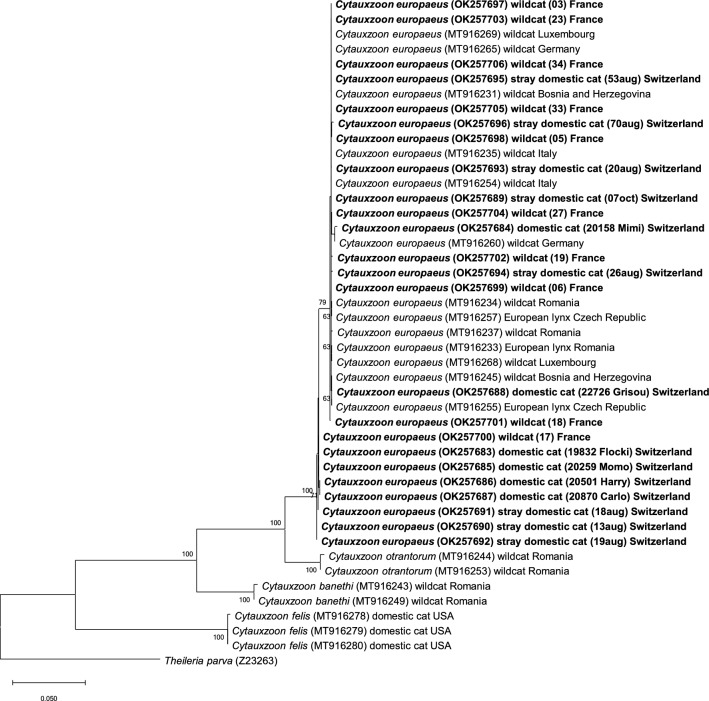
Molecular phylogenetic analysis by maximum likelihood method of the mitochondrial gene *COI*. The evolutionary history was inferred by using the maximum likelihood method based on the Kimura 2-parameter model [54]. The tree with the highest log likelihood (− 5109.71) is shown. The percentage of trees in which the associated taxa clustered together is shown next to the branches (values < 60% are not shown). Initial tree(s) for the heuristic search were obtained automatically by applying the neighbour-joining and BioNJ algorithms to a matrix of pairwise distances estimated using the MCL approach, and then selecting the topology with superior log likelihood value. The tree is drawn to scale, with branch lengths measured in the number of substitutions per site. Host and country origin of the sequences are indicated. GenBank accession numbers are shown in brackets. Isolates from this study are written in bold font

Sequencing of the mitochondrial genes *CytB* and *COI* was successful in 24 of the 27 PCR-positive samples. Samples with high CT values (> 28) could not be amplified; these included the one PCR-positive domestic cat in the countrywide survey (study B) and the PCR-positive cat sample from 2003 (study D). Sequence identities in the mitochondrial genes among isolates of this study ranged from 98.7% (*CytB*) and 98.6% (*COI*) to 100%, with no clear differences between sequences from different study groups. Overall, the study isolates showed < 62.1% and 78.6% sequence identities in the *CytB* and *COI* genes, respectively, with published *C. felis* isolates (Figs. 3, 4). Among the European *Cytauxzoon* spp. isolates, the mitochondrial genes showed highest sequence identities with those of *Cytauxzoon europaeus* (*CytB*: 98.6–100%; *COI*: 98.6–100%), followed by those of *Cytauxzoon otrantorum* (*CytB*: 89.6–90.2%; *COI*: 94.1–95.1%) and *Cytauxzoon banethi* (*CytB*: 76.1–77.8%; *COI*: 88.1–88.8%).

Phylogenetic analysis based on the *18S* rRNA gene showed that the isolates of this study clustered with other European *Cytauxzoon* spp. isolates from domestic and wild felids, and with *C. manul* from a Pallas’ cat (Fig. 2), and was clearly distinct from the *C. felis* cluster. Phylogenetic analysis based on the *CytB* (Fig. 3) and *COI* genes (Fig. 4) clearly assigned all sequences of this study to *C. europaeus*, which was recently described in wild felids in Europe [34].

## Discussion

*Cytauxzoon* spp. infection has been considered a newly emerging disease in domestic and wild felids in central Europe. Our results contradict this assumption and suggest that *Cytauxzoon* spp. infection has been present in domestic cats and European wildcats in central Europe for more than two decades. *Cytauxzoon* spp. was first reported in domestic cats in Spain in 2004 [25] and in the Iberian lynx in Spain in 2003 [57], and subsequently in domestic cats and wild felids in different countries across Europe, including Switzerland [22–24, 26–33, 35]. These *Cytauxzoon* spp. isolates were most closely related to *C. manul,* which was identified in 2005 in a Pallas’ cat imported from Mongolia to Oklahoma [37]. Our results show that *Cytauxzoon* spp. can be detected in European wildcats in eastern France in 1995–1996 and in the DNA of an EDTA-anticoagulated blood sample collected from a domestic cat in central Switzerland in 2003. It is therefore likely that *Cytauxzoon* spp. was already present in domestic cats and wild felids in the decades before the initial report but was overlooked due to its low pathogenic potential compared to *C. felis*.

All *18S* rRNA gene sequences obtained in this study showed high sequence identities and a close phylogenetic relationship, confirming that all isolates belong to the European *Cytauxzoon* spp. cluster regardless of their origin and are distinct from the *C. felis* group. Sequencing of the mitochondrial genes *CytB* and *COI* and differentiation of three different species have recently been documented in European wild felids infected with *Cytauxzoon* spp. in Germany, Italy, Romania, Czech Republic, Bosnia and Herzegovina and Luxembourg [34]. The present study performed these analyses for the first time in *Cytauxzoon* spp.-infected domestic cats in Europe and revealed that all successfully amplified isolates belonged to *C. europaeus*, which was the most common species in European wild felids in the above-mentioned study [34]. Our results revealed that all isolates of *C. europaeus* in the present study shared high sequence identities in the *18S* rRNA, *CytB* and *COI* gene sequences among each other. This is remarkable considering that isolates from both European wildcats and domestic cats were included and that the European wildcat samples originated from collections made in 1995–1996. The close genetic relationship of these isolates supports a potential exchange of *C. europaeus* between domestic and wild felids and thus a potential role of wild felids as reservoirs for this species. Northwestern and western Switzerland, both documented hotspot regions for *Cytauxzoon* spp. infections, harbour stable populations of European wildcats and the Eurasian lynx [58, 59]. Bobcats serve as a major reservoir of *C. felis* in the USA [3]; it is therefore possible that the Eurasian lynx might also play a role as a reservoir of *Cytauxzoon* spp. in central Europe. In support of this possibility, infection with *Cytauxzoon* spp. has been detected in Eurasian lynx in Switzerland (MLM, personal communication). The role of the European wildcat as a reservoir of *Cytauxzoon* spp. is less clear. European wildcats can live in close proximity to and even interbreed with domestic cats [60], which could favour a potential transmission of *Cytauxzoon* spp. between domestic and wild felids. Testing of recent samples from European wildcats in Switzerland indicates that *Cytauxzoon* spp. infections are very frequently detected (MLM, personal communication); however, recent samples from animals from eastern France were not available. The pathogenic potential of *C. europaeus* in European wildcats is unknown. All wildcats included in the present study had been free ranging prior to being hit by a car [41]; thus, no clinical data were available. However, the high prevalence of *C. europaeus* infection in this free-ranging population of European wildcats argues against a high pathogenic potential in this species [31, 32, 34].

The overall prevalence of *Cytauxzoon* spp. infection in samples collected from Swiss domestic cats between 2013 and 2016 was low (0.1%). The one cat whose PCR test result was positive for *Cytauxzoon* spp. in the countrywide survey originated from the canton of Jura in western Switzerland, a previously described hotspot region in which four out of five documented *Cytauxzoon* spp. infections had occurred in domestic cats [29]. In contrast, a remarkably high frequency of *Cytauxzoon* spp. infections was identified in two households in central Switzerland (46.1%) and in stray cats in northwest Switzerland (8.8%). Stray cats live outdoors and are frequently exposed to potential vectors of *Cytauxzoon* spp., such as ticks. An association between infection with *Cytauxzoon* spp. and living in rural areas and outdoor access has recently been documented in domestic cats in Spain and Italy [23, 28]. Similarly, the six *Cytauxzoon* spp.-infected cats identified in the two households in the canton of Aargau in central Switzerland also lived in a rural area, had outdoor access and interacted aggressively with each other. Accordingly, the prevalence of FIV infection in these cats was remarkably high compared with the generally low prevalence of FIV in Switzerland. Of note, the one cat found to be infected with *Cytauxzoon* spp. in study C was also found to be FIV positive. Two cats from household 1 died from bacterial diseases that could have developed as a consequence of aggressive interactions, namely cellulitis on one limb and pyothorax (actinomycosis/nocardiosis). Whether aggressive interactions play a role in the transmission of *Cytauxzoon* spp*.* is unknown. However, for the transmission of *Cytauxzoon* spp., blood-to-blood contact would be required, which is unlikely during cat fights. We have recently documented transmission of *Cytauxzoon* spp. by blood transfusion, but the question of whether blood transfusion from asymptomatic cats can initiate a full cycle of infection in the recipient remains to be elucidated [29].

Most of the cats in this study which were infected with *Cytauxzoon* spp. showed no obvious signs of disease. The schizogonous phase of *C. felis* has been associated with the development of severe clinical disease in domestic cats [3], but schizonts have never been documented in European domestic cats or wild felids infected with *Cytauxzoon* spp. [22, 23, 27]. In the necropsied cat from household 1, we observed very rare enlarged, vacuolated monocytes in capillaries as well as a few aggregates of similarly enlarged macrophages within alveoli that were also strongly CD18 positive, as previously reported for *C. felis* [44]; considering that they were observed in organs that had tested PCR positive for *Cytauxzoon* spp., these results could be interpreted with caution as monocytes/macrophages enlarged by schizonts. To confirm this interpretation, further methodological in situ approaches, such as an in situ hybridization with probes specific for *Cytauxzoon* spp. would be required [61].

The present study also expands the geographic range of *Cytauxzoon* spp. and provides new insights into the frequency of *Cytauxzoon* spp. infection in domestic cats in Switzerland. In a previous study we documented the canton of Jura, close to the French border in the northwest of Switzerland, as a hotspot of *Cytauxzoon* spp. infection, where four out of five cats infected with *Cytauxzoon* spp. originated and three of the five cats were siblings from a stray queen [29]. The second infection hotspot was located in central Switzerland (canton of Aargau) and comprised two neighbouring multi-cat households. These results suggest that *Cytauxzoon* spp. hotspots exist in different parts of the country. Of note, the cat sample from 2003 that tested positive for *Cytauxzoon* spp. also came from this canton, with the sites of origin only 30 km apart. In study C, one infected cat was documented in northern Switzerland, in the canton of Basel-Land, close to the German border. Infection with *Cytauxzoon* spp. was also recently reported in a 6-year-old domestic cat from Saarlouis (Saarland), Germany that presented with anorexia, lethargy and weight loss [30].

Interestingly, *Cytauxzoon* spp. infections were not identified in the eastern and southern part of Switzerland. No samples from stray cats from southern and eastern Switzerland were included in our study, which might have contributed to the lack of *Cytauxzoon* spp. detection in these regions. The geographic differences could also be due to the presence or absence of European wildcats, a potential reservoir of *Cytauxzoon* spp. in Europe. European wildcats are frequently present in the west and northwest regions of Switzerland, but are absent from southern and eastern Switzerland [62]. However, the Eurasian lynx, also a potential reservoir host, is present in all parts of Switzerland [62]. Another explanation for the absence of *Cytauxzoon* spp. infections in eastern and southern Switzerland could be the variable distribution of potential tick vectors within the country. For *C. felis*, *Dermacentor variabilis* and *Amblyomma americanum* ticks are known vectors for transmission [10, 12–14], but neither tick species is present in central Europe. *Ixodes ricinus* is by far the most common tick species in Switzerland and present in all parts of the country [62], whereas *D. marginatus* and *Haemaphysalis punctata* are mainly reported in southern and western Switzerland [62]. *Dermacentor reticulatus* and *Rhipicephalus sanguineus* ticks are rare and mainly found in western Switzerland [62]. The identification of the vector tick species for *Cytauxzoon* spp. in Europe is of pivotal interest to further elucidate the distribution and life-cycle of this pathogen in domestic and wild felids.

## Conclusions

Our study raises the question of whether *Cytauxzoon* spp. is newly emerging in central Europe given that a large proportion of the European wildcat samples collected in 1995 and 1996 in France and a sample from a domestic cat in Switzerland collected in 2003 tested positive for this organism. Mitochondrial gene sequencing revealed that all successfully sequenced *Cytauxzoon* spp. isolates from domestic cats and European wildcats belonged to *C. europaeus*, which was recently shown to be the most common *Cytauxzoon* spp. species in European wild felids. The overall prevalence of *Cytauxzoon* spp. infection in domestic cats in Switzerland was found to be low, but a high prevalence was observed in stray cats and in privately owned cats in hotspot areas, i.e. northwest and central Switzerland. Future studies should address potential vectors and the life-cycle of *Cytauxzoon* spp. in domestic and wild felids in Europe.

## Supplementary Information

**Additional file 1: Table S1.** Origin, signalment, health status, retrovirus status and *Cytauxzoon* spp. results in the 13 domestic cats investigated in study A.

**Additional file 2: Table S2.** Haematological analyses of 5 cats infected with *Cytauxzoon* spp. from households 1 and 2. Results outside the reference interval are shown in bold font.

**Additional file 3: Table S3.** Biochemistry analyses of 5 cats infected with *Cytauxzoon* spp. from households 1 and 2. Results outside the reference interval are shown in bold font.

**Additional file 4**: **Figure S1.** *Cytauxzoon* spp.-positive cat from household 1 (study A), euthanised due to pyothorax consistent with actinomycosis/nocardiosis. Histological and immunohistological features indicative of monocytes/macrophages with schizonts. **a** Lung. Left: focal aggregate of strongly CD18-positive large macrophages in an alveolus (arrow); right: capillary with strongly CD18-positive large monocyte (arrowhead). Alveolar macrophages are also CD18-positive. Immunohistology, haematoxylin counterstain. Bars: 20 µm. **b** Evidence of large vacuolated monocytes. Kidney, glomerulum with structure indicative of large vacuolated monocyte. Haematoxylin eosin stain. Bar: 20 µm. Right insert: Giemsa-stained section of a glomerulum with structure indicative of large vacuolated monocyte. Bar: 10 µm. Left inset: myocardial vessel with structure indicative of large vacuolated monocyte. Haematoxylin eosin stain. Bar: 10 µm.

**Additional file 5: Table S4.** Origin, signalment, health status and retrovirus status of the *Cytauxzoon* spp.-infected domestic cats in studies B–D.

**Additional file 6: Table S5.** Origin, signalment and retrovirus status of the *Cytauxzoon* spp.-infected European wildcats in study D.

## Data Availability

The datasets supporting the conclusions of this article are included within the article. Nucleotide sequences obtained in this study have been submitted to GenBank: *18S* rRNA genes (MW727381–MW727407), *CytB* genes (OK257707–OK257730) and *COI* genes (OK257683–OK257706).

## Abbreviations

*COI*: Cytochrome *c* oxidase subunit I
CT: Cycle threshold
*CytB*: Cytochrome *b*
EDTA: Ethylenediaminetetraacetic acid
ELISA: Enzyme-linked immunosorbent assay
FcaGHV1: *Felis catus* gammaherpesvirus 1
FeLV: Feline leukaemia virus
FIV: Feline immunodeficiency virus
rRNA: Ribosomal RNA
TNA: Total nucleic acid
