# Supplementary material for: Cytauxzoon europaeus infections in domestic cats in Switzerland and in European wildcats in France: a tale that started more than two decades ago
Source: Parasit Vectors. 2022 Jan 8;15:19. doi: 10.1186/s13071-021-05111-8 (PMC8742954; doi:10.1186/s13071-021-05111-8)
Supplement: Supplementary file 1 — Additional file 1: Table S1. Origin, signalment, health status, retrovirus status and Cytauxzoon spp. results in the 13 domestic cats investigated in study A. [file 13071_2021_5111_MOESM1_ESM.docx]

**Additional file 1: Table S1.** Origin, signalment, health status, retrovirus status and *Cytauxzoon* spp. results in the 13 domestic cats investigated in study A

| **House-hold** | **Date of collection** | **Place of origin** | **Canton of origin** | **Country of origin** | **Breed** | **Sex** | **Age (years)** | **Health status** | **FeLV provirus status** | **FIV status^a^** | ***Cytauxzoon* spp. real-time qPCR result (CT values)** | **Accession numbers (sequence length, bp)** | | |
| --- | --- | --- | --- | --- | --- | --- | --- | --- | --- | --- | --- | --- | --- | --- |
|  |  |  |  |  |  |  |  |  |  |  |  | ***18S* rRNA** | ***CytB*** | ***COI*** |
| 1 | Feb 2019 | Unterkulm | AG | Switzer-land | DSH | mc | 5 | Cellulitis of the right forelimb, euthanasia | Negative | **Positive** | **Positive (12.2)** | MW727381 (1591) | OK257730  (1325) | OK257683  (1867) |
| 1 | Mar 2019 | Unterkulm | AG | Switzer-land | DSH | fc | 15 | Unremarkable besides some teeth problems | Negative | **Positive** | **Positive (22.7)** | MW727382 (1565) | OK257729  (1342) | OK257684  (1861) |
| 1 | Mar 2019 | Unterkulm | AG | Switzer-land | DSH | mc | 2 | Unremarkable, developed pyothorax in May 2019 and was euthanised | Negative | Questionable | **Positive (21.0)** | MW727383 (1563) | OK257728  (1331) | OK257685  (1864) |
| 2 | April 2019 | Unterkulm | AG | Switzer-land | DSH | mc | 12 | Unremarkable | Negative | **Positive** | **Positive (22.5)** | MW727384 (1591) | OK257727  (1332) | OK257686  (1870) |
| 2 | May 2019 | Unterkulm | AG | Switzer-land | DSH | mc | 3 | Unremarkable | Negative | Questionable | **Positive (24.4)** | MW727385 (1540) | OK257728  (1341) | OK257687  (1867) |
| 2 | May 2019 | Unterkulm | AG | Switzer-land | DSH | mc | 5 | Unremarkable | Negative | Questionable | **Positive (28.6)** | MW727386 (220) | NS | NS |
| 2 | May 2019 | Unterkulm | AG | Switzer-land | DSH | mc | 12 | Diabetes mellitus | Negative | Questionable | Negative | NA | NA | NA |
| 2 | May 2019 | Unterkulm | AG | Switzer-land | DSH | mc | 4 | Unremarkable | Negative | Questionable | Negative | NA | NA | NA |
| 2 | May 2019 | Unterkulm | AG | Switzer-land | DSH | mc | 3 | Unremarkable | Negative | Questionable | Negative | NA | NA | NA |
| 2 | May 2019 | Unterkulm | AG | Switzer-land | DSH | mc | 4 | Unremarkable | Negative | Questionable | Negative | NA | NA | NA |
| 2 | May 2019 | Unterkulm | AG | Switzer-land | DSH | mc | 4 | Unremarkable | Negative | Questionable | Negative | NA | NA | NA |
| 2 | May 2019 | Unterkulm | AG | Switzer-land | DSH | mc | 10 | Eye problem | Negative | **Positive** | Negative | NA | NA | NA |
| 2 | May 2019 | Unterkulm | AG | Switzer-land | DSH | mc | 10 | Unremarkable | **Positive** | Questionable | Negative | NA | NA | NA |

Abbreviations: DSH, domestic shorthair cat; f, female intact; m, male intact; mc, male castrated; NS, no sequence; NA, not applicable. ^a^ Questionable FIV result: only one band in Western Blot (p24).
