# Supplementary material for: Cytauxzoon europaeus infections in domestic cats in Switzerland and in European wildcats in France: a tale that started more than two decades ago
Source: Parasit Vectors. 2022 Jan 8;15:19. doi: 10.1186/s13071-021-05111-8 (PMC8742954; doi:10.1186/s13071-021-05111-8)
Supplement: Supplementary file 2 — Additional file 2: Table S2. Haematological analyses of 5 cats infected with Cytauxzoon spp. from households 1 and 2. Results outside the reference interval are shown in bold font. [file 13071_2021_5111_MOESM2_ESM.docx]

**Additional file 2: Table S2.** Haematological analyses of five cats infected with *Cytauxzoon* spp. from households 1 and 2^a^. Results outside the reference interval are shown in bold font

| **Household** | **Signalment (breed, sex, age)** | **Health status** | **Date of blood collection** | **Haematocrit (RI) (%)** | **MCV (RI) (fL)** | **MCHC (RI) (g/L)** | **Reticulocytes (x10^9^/L)** | **Leucocytes (RI) (x10^9^/L)** | **Platelets (RI) (x10^9^/L)** |
| --- | --- | --- | --- | --- | --- | --- | --- | --- | --- |
| 1 | DSH, mc, 5 years | Phlegmon of the right frontal leg | Feb 2019^b^ | **23** (28–53) | 46 (39–56) | 18 (18–24) | 9 (≤ 60) | **52** (4–19) | **86** (155–641) |
| 1 | DSH, fc, 15 years | Healthy | Mar 2019 | 36 (28–53) | 55 (39–56) | 19 (18–24) | 14 (≤ 60) | 8 (4–19) | NA |
| 1 | DSH, mc, 2 years | Healthy | Mar 2019 | 36 (28–53) | 40 (39–56) | 22 (18–24) | 24 (≤ 60) | 11 (4–19) | NA |
|  |  | Pyothorax | May 2019^c^ | 42 (33–45) | **34** (41–49) | **38** (33–36) | NA | 7 (5–13) | 394 (180–680) |
| 2 | DSH, mc, 12 years | Healthy | April 2019 | 30 (28–53) | 49 (39–56) | 21 (18–24) | 28 (≤ 60) | 7 (4–19) | 403 (155–641) |
| 2 | DSH, mc, 3 years | Healthy | May 2019 | 39 (33–45) | 45 (41–49) | 30 (33–36) | NA | **21** (5–13) | 399 (180–680) |

Abbreviations: DSH, domestic shorthair; mc, male castrated; fc, female castrated; RI, reference interval; MCV, mean corpuscular volume. MCHC, mean corpuscular haemoglobin concentration; NA, not available. ^a^ No haematological analysis was available from the sixth infected cat from household 2; ^b^ Blood sample collected two days prior to euthanasia; ^c^ Blood samples collected at the time of euthanasia.
