# Supplementary material for: Cytauxzoon europaeus infections in domestic cats in Switzerland and in European wildcats in France: a tale that started more than two decades ago
Source: Parasit Vectors. 2022 Jan 8;15:19. doi: 10.1186/s13071-021-05111-8 (PMC8742954; doi:10.1186/s13071-021-05111-8)
Supplement: Supplementary file 3 — Additional file 3: Table S3. Biochemistry analyses of 5 cats infected with Cytauxzoon spp. from households 1 and 2. Results outside the reference interval are shown in bold font. [file 13071_2021_5111_MOESM3_ESM.docx]

**Additional file 3: Table S3.** Biochemistry analyses of five cats infected with *Cytauxzoon* spp. from households 1 and 2^a^. Results outside the reference interval are shown in bold font

| **House-hold** | **Signalment (breed, sex, age)** | **Date of blood collection** | **Bilirubin (RI) (µmol/L)** | **Urea (RI) (mmol/L)** | **Creatinine (RI) (µmol/L)** | **Total protein (RI) (g/L)** | **Albumin (RI) (g/L)** | **AP (RI) (IU)** | **ALT (RI) (IU)** | **AST (RI) (IU)** | **Potassium (RI) (mmol/L)** | **Chloride (RI) (mmol/L)** |
| --- | --- | --- | --- | --- | --- | --- | --- | --- | --- | --- | --- | --- |
| 1 | DSH, mc, 5 years | Feb 2019^b^ | 4.1 (0–4.6) | 10 (5–11) | 104 (0–203) | 69 (60–84) | **23** (25–39) | 9 (0–72) | **126** (0–91) | **516** (0–49) | 4.4 (3.7–5.7) | 121 (112–124) |
| 1 | DSH, fc, 15 years | Mar 2019 | 3.6 (0–4.6) | 9 (5–11) | 106 (0–203) | **93** (60–84) | 31 (25–39) | 35 (0–72) | 30 (0–91) | 22 (0–49) | 4.1 (3.7–5.7) | 118 (112–124) |
| 1 | DSH, mc, 2 years | Mar 2019 | 3.5 (0–4.6) | 11 (5–11) | 99 (0–203) | 77 (60–84) | 36 (25–39) | 36 (0–72) | 82 (0–91) | **54** (0–49) | 4.3 (3.7–5.7) | 119 (112–124) |
|  |  | May 2019^c^ | **32** (0–3.5) | **14** (7–13) | 107 (98–163) | 64 (64–80) | **27** (32–42) | 6 (0–43) | 37 (0–98) | NA | **5.6** (3.8–5.4) | **107** (113–123) |
| 2 | DSH, mc, 12 years | April 2019 | NA | 8 (5–11) | 119 (0–203) | **96** (60–84) | 26 (25–39) | 24 (0–72) | 31 (0–91) | 24 (0–49) | 4.4 (3.7–5.7) | 118 (112–124) |
| 2 | DSH, mc, 3 years | May 2019 | <2.5 (0–3.5) | 10 (7–13) | **91** (98–163) | 75 (64–80) | 33 (32–42) | 26 (0–43) | 36 (0–98) | 22 (19–44) | 4.4 (3.8–5.4) | 118 (113–123) |

Abbreviations: DSH, domestic shorthair; mc, male castrated; fc, female castrated; RI, reference interval; NA, not available; AP, alkaline phosphatase; ALT, alanine aminotransferase; AST, aspartate aminotransferase. ^a^ No biochemistry analysis was available from the sixth infected cat from household 2; ^b^ Blood sample collected two days prior to euthanasia; ^c^ Blood samples collected at the time of euthanasia.
