# Supplementary material for: Cytauxzoon europaeus infections in domestic cats in Switzerland and in European wildcats in France: a tale that started more than two decades ago
Source: Parasit Vectors. 2022 Jan 8;15:19. doi: 10.1186/s13071-021-05111-8 (PMC8742954; doi:10.1186/s13071-021-05111-8)
Supplement: Supplementary file 4 — Additional file 4: Figure S1. Cytauxzoon spp.-positive cat from household 1 (study A), euthanised due to pyothorax consistent with actinomycosis/nocardiosis. Histological and immunohistological features indicative of monocytes/macrophages with schizonts. a Lung. Left: focal aggregate of strongly CD18-positive large macrophages in an alveolus (arrow); right: capillary with strongly CD18-positive large monocyte (arrowhead). Alveolar macrophages are also CD18-positive. Immunohistology, haematoxylin counterstain. Bars: 20 µm. b Evidence of large vacuolated monocytes. Kidney, glomerulum with structure indicative of large vacuolated monocyte. Haematoxylin eosin stain. Bar: 20 µm. Right insert: Giemsa-stained section of a glomerulum with structure indicative of large vacuolated monocyte. Bar: 10 µm. Left inset: myocardial vessel with structure indicative of large vacuolated monocyte. Haematoxylin eosin stain. Bar: 10 µm. [file 13071_2021_5111_MOESM4_ESM.docx]

**Additional file 4: Figure S1.** *Cytauxzoon* spp.-positive cat from household 1 (study A), euthanised due to pyothorax consistent with actinomycosis/nocardiosis. Histological and immunohistological features indicative of monocytes/macrophages with schizonts. **a** Lung. Left: focal aggregate of strongly CD18-positive large macrophages in an alveolus (arrow); right: capillary with strongly CD18-positive large monocyte (arrowhead). Alveolar macrophages are also CD18-positive. Immunohistology, haematoxylin counterstain. Bars: 20 µm. **b** Evidence of large vacuolated monocytes. Kidney, glomerulum with structure indicative of large vacuolated monocyte. Haematoxylin eosin stain. Bar: 20 µm. Right insert: Giemsa-stained section of a glomerulum with structure indicative of large vacuolated monocyte. Bar: 10 µm. Left inset: myocardial vessel with structure indicative of large vacuolated monocyte. Haematoxylin eosin stain. Bar: 10 µm.

**
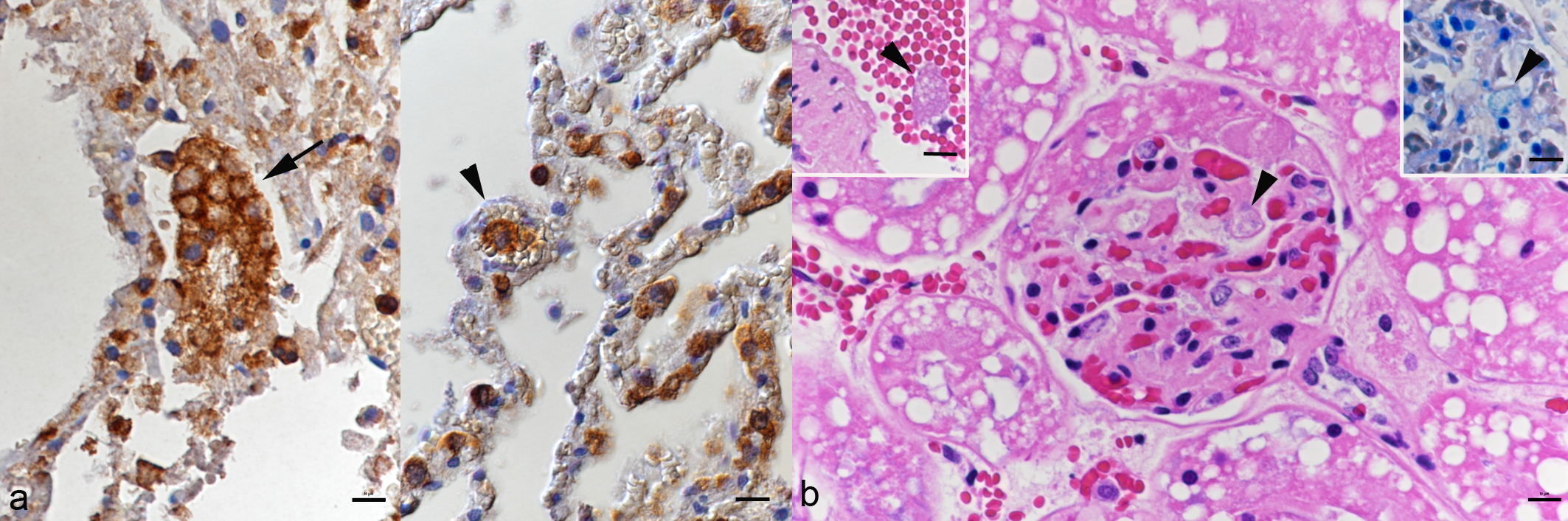
**
