# Supplementary material for: Cytauxzoon europaeus infections in domestic cats in Switzerland and in European wildcats in France: a tale that started more than two decades ago
Source: Parasit Vectors. 2022 Jan 8;15:19. doi: 10.1186/s13071-021-05111-8 (PMC8742954; doi:10.1186/s13071-021-05111-8)
Supplement: Supplementary file 5 — Additional file 5: Table S4. Origin, signalment, health status and retrovirus status of the Cytauxzoon spp.-infected domestic cats in studies B–D. [file 13071_2021_5111_MOESM5_ESM.docx]

**Additional file 5: Table S4**. Origin, signalment, health status and retrovirus status of the *Cytauxzoon* spp.–infected domestic cats in studies B–D

| **Study** | **Date of collection** | **City of origin** | **Canton of origin^&^** | **Country of origin** | **Breed** | **Sex** | **Life-style** | **Age (years)** | **Health status** | **FeLV status** | **FIV status** | ***Cytauxzoon* spp. real-time qPCR result (CT values)** | **Accession numbers (sequence length, bp)** | | |
| --- | --- | --- | --- | --- | --- | --- | --- | --- | --- | --- | --- | --- | --- | --- | --- |
|  |  |  |  |  |  |  |  |  |  |  |  |  | ***18S* rRNA** | ***CytB*** | ***COI*** |
| B | 2015 | Miécourt | JU | Switzer-land | DSH | mc | Privately owned | 11 | Unknown | Provirus negative | NA | 24.5 | MW727389 (1521) | NS | NS |
| C | 2019 | Birsfeld | BL | Switzer-land | DSH | mc | Privately owned | 11 | Severe anaemia and chronic kidney disease | Provirus negative | **Positive** | 14.4 | MW727387 (1538) | OK257725  (1326) | OK257688  (1860) |
| C | 2014 | Alle | JU | Switzer-land | DSH | f | Stray | Unknown | Unknown | Provirus negative | NA | 26.3 | MW727391 (1448) | OK257723  (1334) | OK257690  (1843) |
| C | 2014 | Alle | JU | Switzer-land | DSH | m | Stray | Unknown | Unknown | Provirus negative | NA | 25.6 | MW727392 (1473) | OK257722  (1339) | OK257691  (1873) |
| C | 2014 | Alle | JU | Switzer-land | DSH | m | Stray | Unknown | Unknown | Provirus negative | NA | 33.1 | MW727393 (219) | OK257721  (1333) | OK257692  (1879) |
| C | 2014 | Alle | JU | Switzer-land | DSH | m | Stray | Unknown | Unknown | Provirus negative | NA | 23.5 | MW727394 (1462) | OK257720  (1338) | OK257693  (1866) |
| C | 2014 | Vermes | JU | Switzer-land | DSH | f | Stray | Unknown | Unknown | Provirus negative | NA | 26.6 | MW727395 (1470) | OK257719  (1338) | OK257694  (1547) |
| C | 2014 | St-Ursanne | JU | Switzer-land | DSH | f | Stray | Unknown | Unknown | Provirus negative | NA | 25.5 | MW727396 (1466) | OK257718  (1344) | OK257695  (1867) |
| C | 2014 | St-Brais | JU | Switzer-land | DSH | f | Stray | Unknown | Unknown | Provirus negative | NA | 23.7 | MW727397 (1443) | OK257717  (1334) | OK257696  (1866) |
| C | 2014 | unknown | JU | Switzer-land | DSH | m | Stray | Unknown | Unknown | Provirus negative | NA | 27.2 | MW727390 (1463) | OK257724  (1337) | OK257689  (1852) |
| D | 2003 | Würenlingen | AG | Switzer-land | DSH | mc | Privately owned | 11 | Hepatic lipidosis, icterus, anaemia | p27 negative | Negative | 34.9 | MW727388 (219) | NS | NS |

Abbreviations: AG, Aargau; BL, Basel-Land; JU, Jura; DSH, domestic shorthair; f, female intact; m, male intact; mc, male castrated; NS, no sequence.
