# Supplementary material for: Cytauxzoon europaeus infections in domestic cats in Switzerland and in European wildcats in France: a tale that started more than two decades ago
Source: Parasit Vectors. 2022 Jan 8;15:19. doi: 10.1186/s13071-021-05111-8 (PMC8742954; doi:10.1186/s13071-021-05111-8)
Supplement: Supplementary file 6 — Additional file 6: Table S5. Origin, signalment and retrovirus status of the Cytauxzoon spp.-infected European wildcats in study D. [file 13071_2021_5111_MOESM6_ESM.docx]

**Additional file 6: Table S5.** Origin, signalment and retrovirus status of the *Cytauxzoon* spp.–infected European wildcats in study D

| **Study** | **Date of collection** | **City of origin** | **Department of origin** | **Country of origin** | **Sex** | **Age (morpho-logically)** | **FeLV status** | **FIV status** | **Accession numbers (sequence length, bp)^a^** | | |
| --- | --- | --- | --- | --- | --- | --- | --- | --- | --- | --- | --- |
|  |  |  |  |  |  |  |  |  | ***18S* rRNA** | ***CytB*** | ***COI*** |
| D | 1995–1996 | Richebourg | Haute-Marne | France | f | Adult | **p27 positive** | Negative | MW727398 (1554) | OK257716  (1336) | OK257697  (1850) |
| D | 1995–1996 | Châtenois | Vosges | France | m | Immature | **p27 positive** | Negative | MW727399 (219) | OK257715  (1344) | OK257698  (1871) |
| D | 1995–1996 | Villemaur-sur-Vanne | Aube | France | f | Adult | **p27 positive** | Negative | MW727400 (219) | OK257714  (1335) | OK257699  (1845) |
| D | 1995–1996 | Gye | Meurthe-et-Moselle | France | f | Juvenile | **p27 positive** | Negative | MW727401 (219) | OK257713  (1337) | OK257700  (1862) |
| D | 1995–1996 | Magnant | Aube | France | m | Immature | **p27 positive** | Negative | MW727402 (1367) | OK257712  (1335) | OK257701  (1843) |
| D | 1995–1996 | Champignol-lez-Mondeville | Aube | France | m | Immature | **p27 positive** | NA | MW727403 (216) | OK257711  (1342) | OK257702  (1849) |
| D | 1995–1996 | Beauchemin | Haute-Marne | France | m | Subadult | p27 negative | Negative | MW727404 (1552) | OK257710  (1302) | OK257703  (1548) |
| D | 1995–1996 | Beauchemin | Haute-Marne | France | f | Adult | **p27 positive** | Negative | MW727405 (219) | OK257709  (1348) | OK257704  (1867) |
| D | 1995–1996 | Saint-Dizier | Haute-Marne | France | m | Juvenile | p27 negative | Negative | MW727406 (1573) | OK257708  (1340) | OK257705  (1852) |
| D | 1995–1996 | Mardor | Haute-Marne | France | f | Juvenile | **p27 positive** | Negative | MW727407 (219) | OK257707  (1239) | OK257706  (1547) |

Abbreviations: f, female intact; m, male intact; mc, male castrated; NA, not available. ^a^ The European wildcat samples were only tested by conventional PCR and thus no CT values are available.
